# Leveraging AI to Investigate Child Maltreatment Text Narratives: Promising Benefits and Addressable Risks

**DOI:** 10.2196/73579

**Published:** 2025-07-24

**Authors:** Wilson Lukmanjaya, Tony Butler, Sarah Cox, Oscar Perez-Concha, Leah Bromfield, George Karystianis

**Affiliations:** 1 School of Population Health, University of New South Wales Sydney, F25, Samuels Building, Samuel Terry Ave, Kensington, Sydney, 2033, Australia, 61 293853136, 61 93136185; 2 Australian Center for Child Protection, University of South Australia, Adelaide, Australia; 3 Centre for Big Data Resarch in Health, University of New South Wales Sydney, Sydney, Australia

**Keywords:** artificial intelligence, large language models, child maltreatment narratives, text mining, benefits and risks, research framework

## Abstract

The trove of information contained in child maltreatment narratives represents an opportunity to strengthen the evidence base for policy reform in this area, yet it remains underutilized by researchers and policy makers. Current research into child maltreatment often involves the use of qualitative methodologies or structured survey data that are either too broad or not representative, thereby limiting the development of effective policy responses and intervention strategies. Artificial intelligence (AI) approaches such as large language models (AI models that understand and generate language) can analyze large volumes of child maltreatment narratives by extracting population-level insights on factors of interest such as mental health and treatment needs. However, when applying such methods, it is useful to have a framework on which to base approaches to the data. We propose a seven step framework: (1) data governance; (2) researcher vetting; (3) data deidentification; (4) data access; (5) feasibility testing of baseline methods; (6) large-scale implementation of black box algorithms; and (7) domain expert result validation for such exercises to ensure careful execution and limit the risk of privacy and security breaches, bias, and unreliable conclusions.

## Smarter Tech, Safer Kids

The World Health Organization estimates around 1 in 2 children worldwide experience physical, sexual, and emotional abuse and neglect [1]. In Australia, the monitoring of child maltreatment (ie, “all forms of physical and emotional ill-treatment, sexual abuse, neglect, and exploitation that results in actual or potential harm to the child’s health, development or dignity” [2,3]) relies on administrative data provided by child protection departments for local and national reporting purposes [4-6]. However, the structured nature of these data limits the reporting of more nuanced information such as maltreatment types, treatment needs, and mental health, preventing the ability to answer key questions. For example, the 2021 Australian Child Maltreatment Study [6] recorded the prevalence of physical abuse and sexual abuse at 32.0% and 28.5%, respectively, whereas the Australian Bureau of Statistics [5] in the same year reported that 1 in 7 (14%) children experienced physical and sexual abuse. Several factors contribute to such discrepancies, including the use of different methodologies (eg, self-report, cross-sectional surveys, and surveillance through administrative data), varying definitions of abuse and age ranges, and biases due to the small sample sizes and response rates [7-9]. While high-level reporting is important, both sources fail to provide a comprehensive picture of child maltreatment necessary for effective policy responses. Characteristics such as uncommon maltreatment subtypes and treatment needs are lacking. Indeed, the Australian Productivity Commission states that “with technological developments and advances in analytical techniques, not only is the volume of data being generated and collected growing, but so too is the scope to make use of data in innovative ways in every sphere of life*”* [10]. Child protection should be no exception to this.

Research in child maltreatment has predominantly utilized statistical and artificial intelligence (AI) approaches such as artificial neural networks and predictive risk modeling applied to structured child protection administrative data [11-15]. Studies on health administrative records focused on developing models to predict cases of sexual abuse [11], physical abuse [12], and overall maltreatment [13-15], with robust performance. Furthermore, information has been successfully extracted using text mining techniques such as word embeddings, bag-of-words, and feature selection from free-text health notes [16-19] to detect physical abuse.

Nevertheless, rarely tapped sources of unstructured information in this area are child maltreatment narratives containing detailed descriptive accounts of incidents or experiences of physical or emotional harm to a child. These document the initial reasons for coming to the attention of authorities (ie, intake report), descriptions of a child’s experience of abuse or neglect, psychologists’ assessments, police and medical records, and a detailed history of child protection involvement. They also describe abuse types, substance use and abuse, recorded injuries, mental and behavioral problems, physical health conditions, parental and family characteristics, and the types and conditions of the accommodation. Utilizing the information contained within the narratives could fill existing knowledge gaps by highlighting less common abusive behaviors (eg, social abuse, coercive control) and enriching definitions and measures of abuse types and mental illnesses [20-22]. A limited number of studies have used these narratives to automatically extract information. Victor et al [23] and Perron et al [24,25] applied machine learning algorithms such as random forest and k-nearest neighbors on caseworker summaries to identify domestic violence events and opioid-related maltreatment risks, respectively. In addition, Saxena et al [26] used topic modeling to explore the concept and definitions of “risk” on child maltreatment records with promising performance. Despite the advent of newer technologies to conduct text analysis such as large language models (LLMs; AI models that can understand and generate language), limited attempts have been made [27,28] to implement this approach. In particular, Perron et al [27] reported an *F*_1_-score of 89%‐95% for their model to classify and extract substance-related problems, while more recently, Stoll et al [28] noted their approach to classify maltreatment subtype yielded a superior performance against human reviewers by 10%.

However, automatically mining information from child maltreatment records faces significant limitations [23-28]. Most importantly, the lack of sufficient training data restricts the AI model’s capacity to learn complex patterns [29] such as nuanced indicators of abuse. In addition, the limited contextual understanding (even when using LLMs), along with the inconsistencies in narrative style and language, as caseworkers may document—or omit—events of child maltreatment through various terms, descriptions, and writing style, can lead to inaccurate outputs and falsehoods [26]. This is particularly critical when analyzing data related to Indigenous populations requiring a specific cultural lens through which to examine the data. Relying exclusively on one data type (eg, administrative data) can also skew the results and present an inaccurate picture [30].

## Benefits Versus Risks

To manually process such voluminous and diverse information is extremely time consuming and not without significant challenges, including a lack of human resources to inspect large scale data and the associated high cost. AI pipelines, particularly applications of LLMs, offer a solution for efficiently processing vast amounts of child maltreatment narratives, comprehending the text for a broader range of tasks (ie, summarization, classification) [27,28,31], and offering potentially greater quality control due to its standardized approach compared to other (eg, qualitative) methods [32]. A primary concern of child protection services is to safeguard and monitor those in their care who may be at risk of maltreatment and neglect. Timely processing of many thousands of cases provides the possibility for professionals and policy makers to make faster decisions (eg, identifying clusters of abuse or mental illness using geographic areas) by predicting the severity of future abuse or allocating resources to high-prevalence communities and intervene earlier. Consequently, new evidence-based protocols for early intervention and prevention can be developed aimed at improving outcomes for children and families.

While appealing, AI applications come with security risks (eg, malware, data leakage), performance issues (eg, “AI hallucinations”), technical limitations (eg, black box bias), and ethical conundrums (eg, machine vs human decisions). Hidden malware or malicious code within unvetted AI algorithms with built-in third-party components could result in leaking sensitive information residing in child maltreatment narratives [33,34]. Child protection departments are extremely cautious about providing data access (making this data some of the most difficult to access) as children’s data privacy risks could expose them to further harm. When the data involve Indigenous populations, additional cultural considerations for access, governance, and interpretation are required. Even with data access approval, challenges related to narrative data quality, such as data omission, will persist as researchers have limited control over these factors. Cultural and linguistic biases, as well as selection bias in the training data, also pose risks for misinterpretation of findings. In particular, LLM performance can be vulnerable due to “hallucinations” or misinformation with models generating inaccurate or false conclusions. This is further amplified by black box bias [35-37], since the decision-making process is not visible or understandable by humans and, therefore, it is hard to conduct any detailed error analysis.

For these reasons, AI-generated misinformation can have larger implications due to false positives and negatives, fabrication of summarization, and misclassifications. In child maltreatment cases, this could lead to the misidentification of mental health disorders, abuse and neglect misclassification, and unnecessary family separation [38-42]. Falsehoods could be interpreted as gold standards. Therefore, child maltreatment policies require a decision framework that draws conclusions from both machines and humans, with the final decision relying on human expertise. While some may propose automated approaches can reduce human errors, bias, and the time required to process reports, replacing human decision-making with AI-powered approaches requires ethical protocols to be in place. Relying exclusively on algorithms and their outputs should be discouraged as they lack the complexities and subtleties of human reasoning and decision making [43-47]. Human judgement needs to take priority over AI outputs [48] and adhere to research ethics and legal standards [49-51]. Machine-made choices bear potentially severe consequences when it comes to enhancing the evidence base and policy translation leading to social injustice and population oversurveillance [48]. Considering the inherent risk of biases [52,53] (ie, underrepresented groups sampling bias) and the cost of hardware requirements, security protocols, and manual code inspection, it is difficult to apply such algorithms widely without the appropriate precautions.

Risk mitigation strategies are essential to ensure the appropriate application of automated approaches in areas such as child protection. First, using deidentified data and downloadable models from verified sources minimizes the risk of leaking private information. The incorporation of local LLMs could be set up using a virtual machine and offline to prohibit data access and the possibility of reidentifying personal records if information were to be siphoned off through hidden malware. False outcomes could be mitigated through improved quality of input data, further training to improve the model’s understanding and designing clear, precise instructions for the model (ie, design prompt) that steer its response towards accurate information [47,48]. Governance infrastructures are also necessary to ensure appropriate handling of such sensitive data and the generated findings, especially if these data involve Indigenous populations, which would require input from key stakeholder groups and people with lived experience to ensure culturally sensitive analysis and interpretation. Finally, pipelines need to have expert-in-the-loop architecture to improve the quality and reliability of results. Ensuring that pipelines powered up by LLMs operate transparently with domain experts’ input not only protects against biased decision-making but also aligns with efforts to safeguard personal data, as some algorithms can obscure the processes that may compromise fairness and privacy.

## Proposed Framework

Existing research using AI approaches on child maltreatment narratives follows the establishment of the technical pipeline to extract information and obtain data access. Additional steps (eg, governance, data deidentification, researcher vetting) are also required to not only enhance the performance of such approaches but to limit security risks, ensure translatable outputs, and boost public confidence in such approaches. For these reasons, we propose a seven-step framework for processing child maltreatment narratives with LLMs or any black box algorithm: (1) data governance (including cultural considerations where appropriate), (2) researcher vetting, (3) data deidentification, (4) data access, (5) feasibility testing of baseline methods, (6) large-scale implementation of black box algorithms, and (7) domain expert result validation. These steps align with Gillingham’s [48] principles of algorithmic accountability in social work and existing policy guidelines on AI for children [54,55] that prioritize children’s safety, well-being, data privacy, and AI transparency. Although Gillingham’s principles are rooted in decision support tools, they are relevant due to the generated results potentially contributing to policy making and translation.

The seven steps include:

1. Data governance: this foundational step focuses on establishing appropriate data governance infrastructures on both the child maltreatment data and the generated outputs. Key stakeholder agencies such as departments of child protection and information and communication technology services along with ethics committees must be engaged before any data analysis takes place. If the data relate to Indigenous populations, it is necessary to include relevant community representatives to ensure the analysis interpretation and dissemination of findings are culturally appropriate and align with Indigenous data sovereignty.
2. Researcher vetting: researchers need to be vetted (eg, have no track record of criminal activity, have previous experience with handling and analyzing sensitive data, be domestically based) to ensure responsible research, including a working with children government check. Signing a nondisclosure agreement can further minimize potential risks such as data privacy breaches and identity theft.
3. Data deidentification: as child protection data contains extremely sensitive information (eg, name, addresses, date of birth), to minimize privacy breaches, deidentification of child maltreatment narratives is recommended. Thus, in the unlikely event of data leakage, deidentification ensures that no identifiable information is exposed in the dataset.
4. Data access: accessing child protection data should be done within the vicinity of child protection agencies who can assign researchers limited access and maintain history logs of accessed or modified documents. Such an approach will ensure transparency and provide the capacity for tracking errors and auditability.
5. Feasibility testing of baseline methods: conducting a feasibility test before any large-scale implementation is important. Implementing simpler approaches that rely on lexical pattern identification (eg, rule-based) or traditional machine learning algorithms offers a controlled, auditable environment with enhanced security reducing the risk of unintended data exposure, allowing for more interpretability on potential errors (ie, false positives, false negatives) without requiring an extraneous number of resources and costs [56].
6. Large scale implementation of black box algorithms: the implementation of black box approaches or algorithms ensures efficient and effective large-scale processing of voluminous datasets. Evaluation metrics such as precision and recall ensure robust performance and support scalable research to address knowledge gaps in child maltreatment. Models such as LLMs can assist in the contextual understanding and scalability of the research to ultimately narrow the information gap within specific research aims in child maltreatment. These applications can be applied offline through a secure virtual machine environment. Through this approach, child protection organizations can ensure that researchers have no means of extracting and downloading identifiable data without explicit approval from participating governmental departments.
7. Domain expert result validation: the returned results need to be validated by domain experts in child maltreatment to ensure that the extracted or generated information does not lead to inaccurate or misleading conclusions. This can be done by manually checking a random sample of narratives and conducting interannotator agreement (via metrics such as Cohen κ coefficient) between 2 to 3 experts. Disagreements between domain experts regarding claims may require researchers to retrain and re-evaluate their method. This evaluation and validation process will ensure the findings’ accuracy and relevancy are aligned with ethical standards such as the policy guidance for AI for children [54] or transnational frameworks for AI and children’s rights and well-being [55], ensuring accuracy and fairness and limiting risks of potential harm before being used towards shaping existing or new policies and protocols.

**Figure 1.**
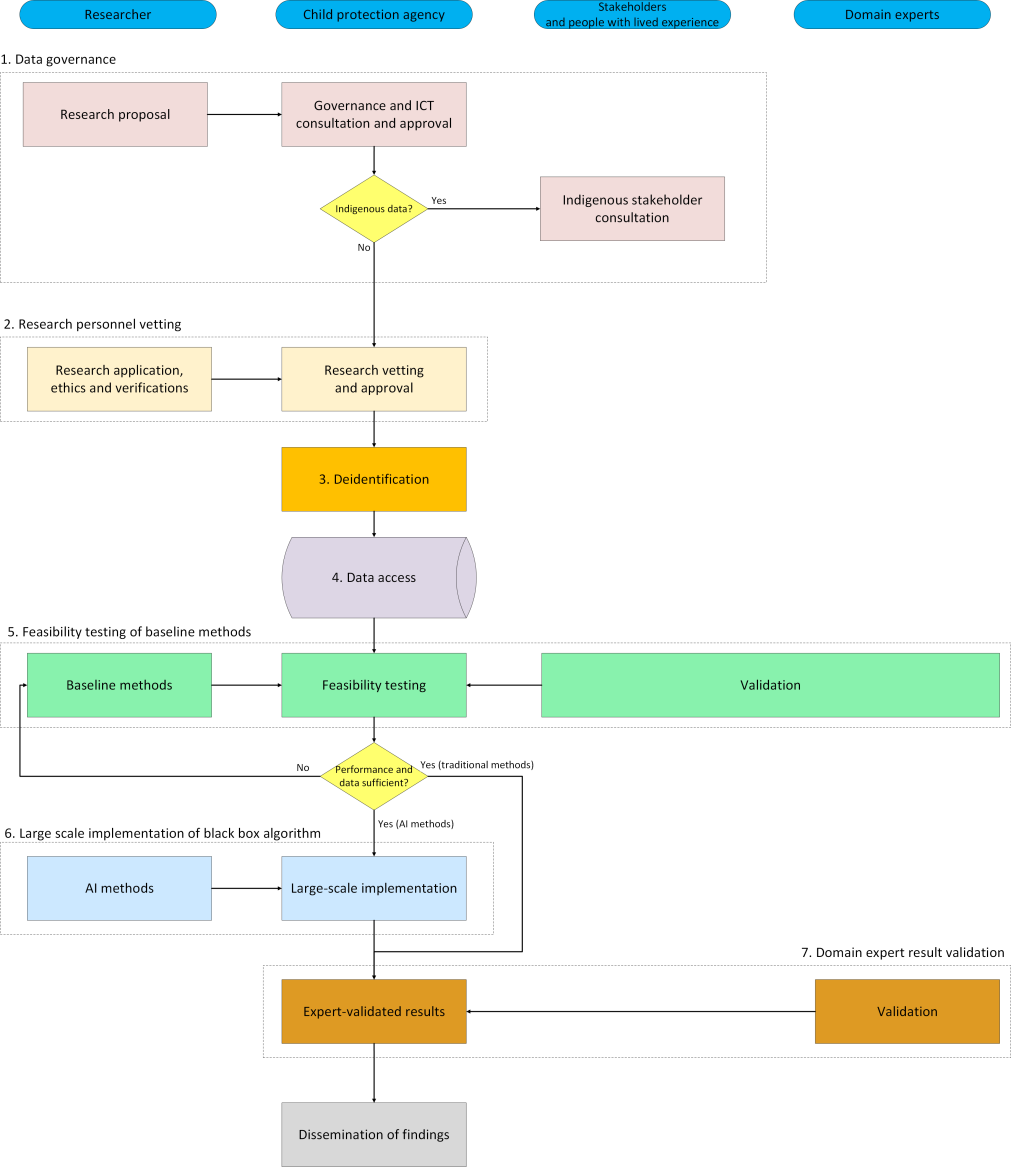
Illustrative example of the proposed 7-step framework for responsible AI use in child maltreatment research. AI: artificial intelligence; ICT: information and communications technology.

Our proposed 7-step framework offers a novel research pipeline specific to ethical and secure use of AI in child maltreatment research. By introducing data governance, the inclusion of stakeholders with lived experience and structured deidentification processes and feasibility testing, the framework prioritizes both ethical and cultural safety while promoting analytical rigor. Incorporating domain expert validation further enhances the interpretability, contextual relevance, and ethical soundness of AI outputs—elements that are largely absent in the current literature. An illustrative example of a brief case scenario is presented in Figure 1. While it is understood that the framework may not apply to all contexts—given that protocols can vary across countries and may not have Indigenous cultures—the core principles are common. Adhering to these principles supports data privacy and cultural sensitivity and helps mitigate the uncaptured context problem and bias mitigation within the field through domain expert result validation [57].

## Conclusions

As the complexity and scale of child maltreatment increases and the volume of information expands, an opportunity exists to apply new and sophisticated approaches that rely on AI to strengthen the evidence base by processing large volumes of narrative data. However, with this opportunity come risks that need to be minimized. The implementation of AI methods, particularly black box methods such as LLMs, should follow a framework of seven key steps—(1) data governance, (2) researcher vetting, (3) data deidentification, (4) data access, (5) feasibility testing of baseline methods, (6) large-scale implementation of black box algorithms, and (7) domain expert result validation—to ensure culturally sensitive governance, minimization of privacy risks, improvement data analysis, and optimization of AI usability in strengthening the evidence base for child maltreatment. Further research is needed to evaluate the framework’s effectiveness and practice to improve policies and lead to better outcomes in child protection.

## Abbreviations

AI: artificial intelligence
LLM: large language model
